# As-Cast Magnesium Alloys with Ca Addition as a Replacement for Magnesium Alloys with Rare Earth Metals

**DOI:** 10.3390/ma18081860

**Published:** 2025-04-18

**Authors:** Tomasz Rzychoń, Agnieszka Fornalczyk

**Affiliations:** 1 Department of Materials Technologies, Faculty of Materials Engineering, Silesian University of Technology, 40-019 Katowice, Poland; tomasz.rzychon@polsl.pl; 2 Department of Production Engineering, Faculty of Materials Engineering, Silesian University of Technology, 40-019 Katowice, Poland

**Keywords:** magnesium alloys, gravity casting, creep properties, microstructure

## Abstract

This article evaluates the possibility of replacing creep-resistant magnesium Mg-Zn-RE-Zr alloys (EZ33) with Mg-Al-Ca-Sr alloys. (1) Background: Mg alloys with RE metals show excellent properties. Due to their high cost, new, more economical Mg alloys are being developed. Replacing RE metals with cheaper elements such as Al and Ca allows us to obtain high mechanical properties at elevated temperatures due to the tendency to form stable intermetallic phases. (2) Methods: Microstructure analysis (LM, SEM, TEM, and XRD) was performed and mechanical properties were tested at ambient and elevated temperatures. (3) Results: Increasing the Ca content and decreasing the Al content leads to the formation of a continuous skeleton of high-melting and brittle Ca-rich Laves phases and Sr-rich intermetallic phases and the formation of plate-like precipitates of the C15 phase inside the α-Mg solid solution. The crystallographic orientation of plate-like precipitates contributes to the blocking of dislocations in slip systems activated at elevated temperatures. Increasing the Ca and Sr content allows for the regulation of the Al concentration in the α-Mg, providing solution strengthening and stability of the α-Mg solid solution. These factors contribute to a significant improvement in creep resistance of Mg-Al-Ca-Sr alloys. (4) Conclusions: The strength properties and elongation at ambient temperature of the Mg alloys with Ca and Sr addition are comparable to those of the EZ33 alloy, and due to the presence of lighter alloying elements, a better specific strength is achieved. Ca-rich Mg-Al-Ca-Sr alloys exhibit better creep resistance at temperatures of up to 200 °C compared to the EZ33 alloy.

## 1. Introduction

Both the aviation and automotive industries show great interest in as-cast magnesium alloys, which are characterized by low density (about 1.8 g/cm^3^), high specific strength, good castability, machinability, weldability in controlled atmospheres, and high vibration damping capacity. The range of applications of magnesium alloys is limited by their low modulus of elasticity, flammability, low plasticity, high susceptibility to electrochemical corrosion, and low creep resistance [1].

As-cast magnesium alloys with aluminum, zinc, and manganese (AZ91, AM50, and AM60) have found the greatest number of industrial applications, especially in the automotive industry [2,3,4,5,6]. A significant disadvantage limiting the wider use of magnesium–aluminum alloys is the low maximum operating temperature under long-term loads, which should not exceed 120 °C. It is due to the presence of the Mg_17_Al_12_ phase, characterized by unfavorable morphology and low melting point. For this reason, in the last two decades, intensive research has been carried out on cast magnesium alloys intended for operation at temperatures higher than 120 °C, which could be used for automatic transmission housings, automotive engine blocks, or engine intake housings in aviation. Creep resistance is improved by incorporating strontium, silicon, calcium, and rare earth metals into Mg-Al alloys or by developing aluminum-free alloys, e.g., Mg-RE-Zr, Mg-Gd-Nd-Zr, Mg-Y-RE-Zr, Mg-Sc-Mn, and Mg-Ag-Nd-Zr. These alloying elements cause the elimination of the low-melting Mg_17_Al_12_ phase and the formation of intermetallic phases characterized by high stability of the crystal structure and morphology at temperatures above 120 °C.

According to the current state of knowledge, assuming the criterion of maximum operating temperature, cast magnesium alloys can be divided into three groups [7,8,9,10,11,12,13,14,15]:

- Common Mg-Al alloys with the addition of zinc or manganese (AZ91, AM50, and AM60), which can be high-pressure- or gravity-cast (operating temperature up to 120 °C);
- Mg-Al-RE (AE44), Mg-Al-Ca (AXM53), Mg-Al-Sr (AJ62), and Mg-Al-Si (AS21) die-cast alloys and Mg-Sr-Mn and Mg-RE-Zn-Zr (EZ33) gravity-cast alloys (operating temperature up to 200 °C);
- Mg-Gd-Nd-Zr (Elektron 21), Mg-Y-RE-Zr (WE43), Mg-Ag-Nd-Zr (QE22), and Mg-Sc-Mn gravity-cast alloys (operating temperature up to 250 °C; alloys containing scandium up to 300 °C).

Among the alloying elements used in magnesium alloys, rare earth metals have the most beneficial effect on creep resistance. However, due to the high cost of rare earth elements, efforts are being made to develop new magnesium alloys that do not contain these elements [16]. Therefore, research is conducted on magnesium alloys with cheap alloying elements, such as Al, Ca, Sr, Si, Mn, Zn, and Sn, in order to obtain Mg alloys with similar mechanical properties at ambient and elevated temperature comparable to those of Mg alloys with rare earth metals [6,17].

An example of a material with good creep resistance up to 200 °C is the EZ33 alloy, which is designed for gravity casting. It has been successfully used in the aviation industry. Meanwhile, it is possible to replace the EZ33 alloy with much cheaper Mg alloys [18,19,20]. It has already been shown that the addition of Ca to Mg-Al alloys significantly contributes to the improvement in creep resistance of die-cast alloys [1]; however, the influence of this element on the mechanical properties of sand castings is poorly understood.

This article compares the microstructure and mechanical properties of gravity-cast Mg-Al-Ca-Sr alloys with commercial sand-cast EZ33 (Mg-Zn-RE-Zr) magnesium alloys and assesses the possibility of replacing the EZ33 alloy with cheaper magnesium alloys consisting of Al and Ca.

## 2. Materials and Methods

The magnesium alloy containing Al, Ca, Sr, and Mn was prepared (Table 1), and its composition was analyzed using a SPECTROMAX optical emission spectrometer (AMETEK, Berwyn, IL, USA). Commercially pure Mg, Al, and Mn were used, and Sr and Ca were added in the form of Al-10 wt% Sr and Al-85 wt% Ca master alloys, respectively. Melting of the alloys was performed by induction melting in an alumina crucible under the protection of an argon atmosphere. The melt was maintained at 730 °C for 3 min and then poured into sand molds. Induction melting was also used to melt EZ33 ingots, but casting was performed at a temperature of 780 °C, in accordance with the manufacturer’s recommendations.

The microstructure of the AXJM series and the EZ33 alloys was analyzed through light microscopy (LM) using an Olympus GX-71 microscope (Olympus, Tokyo, Japan)

Microstructural observations at higher magnification were carried out using an FE SEM Hitachi S-3400N (Hitachi, Tokyo, Japan) scanning electron microscope and Titan 80-300 (FEI, Hilsboro, OR, USA) and Hitachi HD-2300A (Hitachi, Tokyo, Japan) scanning transmission electron microscopes. Chemical composition microanalysis of the intermetallic compounds was performed through energy-dispersive X-ray spectroscopy (EDS) using an EDS Thermo Noran spectrometer (Thermo Fisher Scientific, Waltham, MA, USA). In order to analyze the chemical composition of the α-Mg solid solution and plate-like precipitates of the C15 phase, electron energy-loss spectroscopy (EELS) was performed using a Titan 80-300 (FEI, Hilsboro, OR, USA) microscope.

Thin foils for microscopic observation were prepared using two methods:

- Electrolytic polishing (double-stream) using a Tenupol-5 polisher: The electrolyte used was composed of 5.3 g of lithium chloride, 11.16 g of magnesium perchlorate, 500 mL of methanol, and 100 mL of 2-butoxy-ethanol. Polishing was performed at −45 °C at a voltage of 20 V.
- FIB method using a Quanta 3D 200i device: The following operating parameters were used: resolution in FIB mode at 7 nm at an accelerating voltage of 30 kV, beam current of 1 pA, and magnification range from 50× to 200,000×. SEM imaging was performed with a resolution down to 3 nm at an accelerating voltage of 30 kV in high vacuum (HiVac), 12 nm at an accelerating voltage of 3 kV in low vacuum (LowVac), and 3 nm at an accelerating voltage of 30 kV in environmental mode.

The volume fraction of the phases and grain size was measured through quantitative metallography using METILO 14.1 software (Silesian University of Technology, Katowice, Poland).

XRD patterns were obtained using a Philips X`Pert 3 Powder (Philips, Eindhoven, The Netherlands) diffractometer with a copper anode. Registration was performed by 0.02° stepwise regression for 2θ ranging from 10 to 140°. Phase identification was performed using the ICDD PDF-4+ database (ICDD, Philadelphia, PA, USA). The Rietveld method was used to determine the lattice parameters of the α-Mg phase. The structure models for the α-Mg, Mg_17_Sr_2_, Al_4_Sr, Mg_17_Al_12_, Al_2_Ca, Mg_2_Ca, and Al_1.34_CaMg_0.66_ phases were taken from the ICDD PDF-4+ database.

Tensile and creep tests were performed on a Zwick/Roell Kappa 50DS electromechanical testing machine (ZwickRoell, Ulm, Germany). The creep tests were carried out under constant load in a temperature range between 180 °C and 250 °C and at stresses between 30 and 90 MPa. The creep test conditions were selected based on the temperature and stress levels experienced during the service life of engine hulls used in helicopters. Samples were heated up in a resistance furnace, and the temperature was measured using thermocouples attached to the sample at three positions of the gauge part to maintain the temperature within 3 °C. The creep strain was measured by extensometers, which were attached directly to the gauge section of the specimens. The initial distance between the extensometer arms was 50 mm. The length of the specimen was 100 mm, the gauge length was 60 mm, and the diameter of the reduced section was 6 mm. Short creep tests (100 h or until rupture) were used to evaluate the creep properties of Mg-Al-Ca-Sr alloys. Samples with the same geometry were also used in tensile tests. The strain rate was 4 mm/min, and a contact extensometer was used to measure the extension of a specimen under load. Force and the displacement of extensometer arms was continuously recorded during the test using testXpert II testing software (ZwickRoell, Ulm, Germany).

## 3. Results and Discussion

### 3.1. Microstructure of Mg-Al-Ca-Sr Alloys

**Figure 1:**
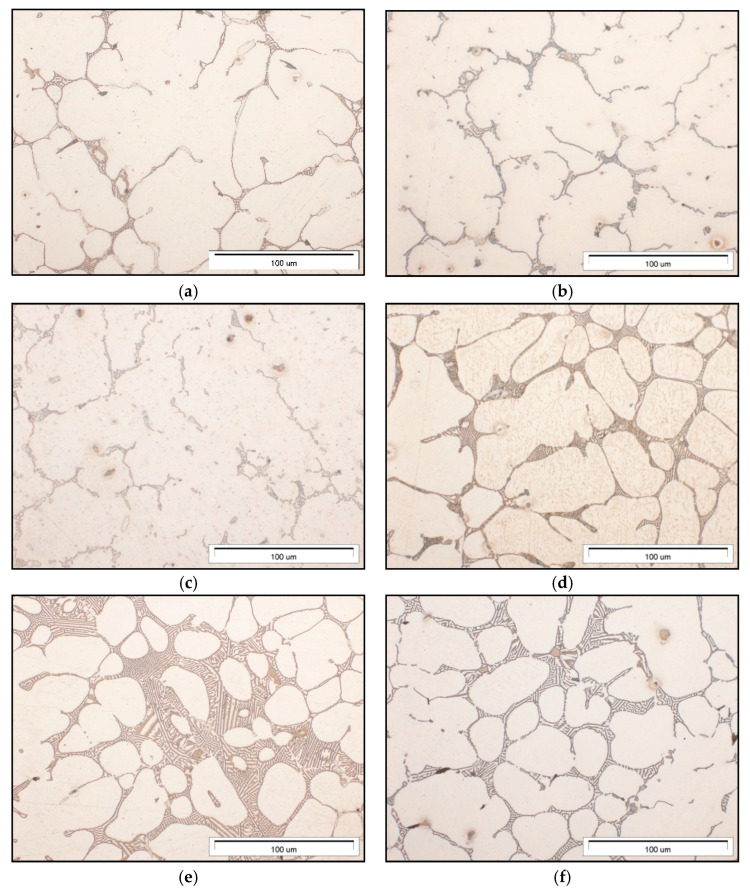
Microstructure of AXJM51 (**a**), AXJM71 (**b**), AXJM91 (**c**), AXJM53 (**d**), AXJM73 (**e**), and AXJM93 (**f**) magnesium alloys.

The microstructure of gravity-cast Mg-Al-Ca-Sr alloys consists of the α-Mg matrix and intermetallic compounds at the interdendritic regions (Figure 1). Additionally, in alloys consisting of 3% Ca, the fine precipitates inside α-Mg were observed. An increase in the content of alloying elements leads to an increase in the content of intermetallic phases and the formation of continuous interdendritic networks.

**Figure 2:**
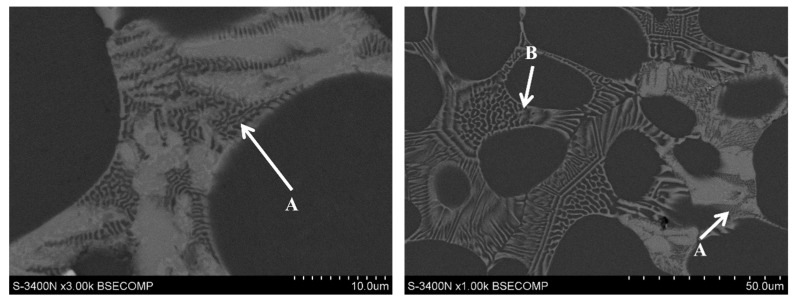
Thin lamellar C14 (A) phase and thick lamellar C36 (B) phase in AXJM53 alloy; SEM.

**Figure 3:**
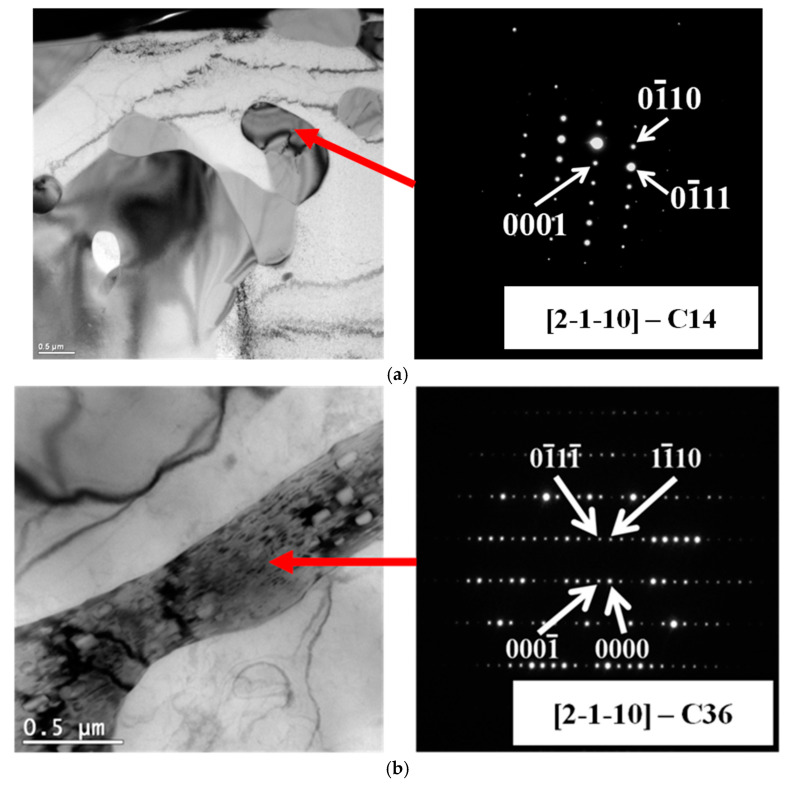
Microscopic images and SAED patterns of C14 phase (**a**) and C15 phase (**b**) obtained by beam parallel to [hki0] axis; B II $[2\overset{-}{1}\overset{-}{1}0]$, TEM, AXJM53 alloy.

The thin lamellar phase (Mg,Al)_2_Ca (marked with the letter A in Figure 2) and the thick lamellar phase (Al,Mg)_2_Ca (letter B in Figure 2) exhibit a similar chemical composition (Table 2) and are characterized by a hexagonal crystal structure of the C14 and C36 type (Figure 3), respectively. The lattice parameters a_0_ of these compounds are comparable, while the lattice parameter c_0_ of the C14 phase is almost twice that of the (Mg,Al)_2_Ca-C36 phase. Therefore, during identification with the use of selected-area electron diffraction on a transmission electron microscope, it is necessary to obtain the appropriate orientation of the thin foil in relation to the incident electron beam, which enables the determination of interplanar distances d_0001_. From a practical point of view, the identification of the C14 and C36 phases can be carried out for an incident electron beam B = hki0. The results of the electron diffraction analysis for both phases are shown in Figure 3.

**Figure 4:**
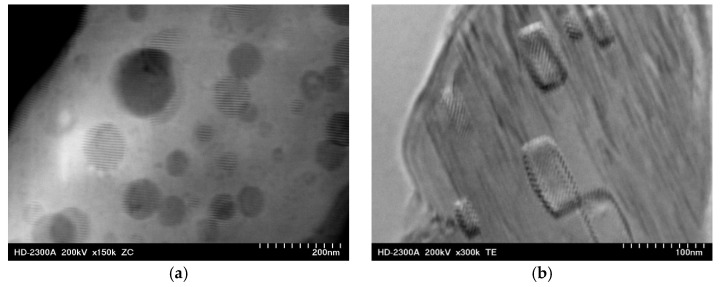
Spherical clusters with moiré fringes observed inside the C36 phase (**a**) and rectangular clusters with moiré fringes observed inside the C36 phase (**b**). STEM, AXJM53 alloy.

In Mg-Al-Ca-Sr alloys containing 1 wt% Ca, the chemical composition of the (Mg,Al)_2_Ca phase with a C36 structure is homogeneous, while in the alloys containing 3 wt% Ca, spherical or rectangular Mg-rich clusters were observed inside the C36-(Mg,Al)_2_Ca phase (Figure 4). These clusters were also observed in Ref. [21], where the islands were classified as α-Mg solid solution, while in [22], it was shown that Mg-rich clusters have the C36 crystal structure due to the large solubility range of Al and Mg in the C36-(Mg,Al)_2_Ca phase. The role of clusters does not seem to have a major impact on the mechanical properties; however, at high temperatures (above 500 °C), they contribute to the formation of cracks inside the C36 phase.

**Figure 5:**
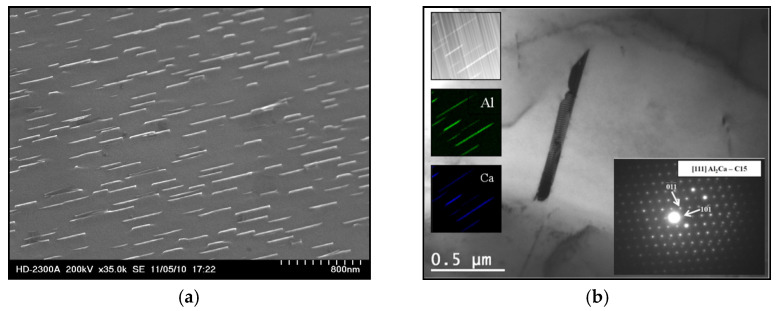
Plate-like precipitates of the Al_2_Ca (C15) phase inside the α-Mg solid solution; AXJM53 alloy. STEM image (**a**); TEM image with the SAED pattern and surface distribution of elements (**b**).

Tiny plate-like precipitates inside the α-Mg solid solution were identified as the Al_2_Ca phase with a cubic C15-type crystal structure (Figure 5), which are characterized by a crystallographic relationship with the {111}_C15_‖(0001)_α-Mg_ $[01\overset{-}{1]}$_C15_ ‖$[0\overset{-}{1}10]$_α-Mg_. It indicates that these precipitates lie in the basic plane of the hexagonal crystal system of the α-Mg solid solution [23,24].

**Figure 6:**
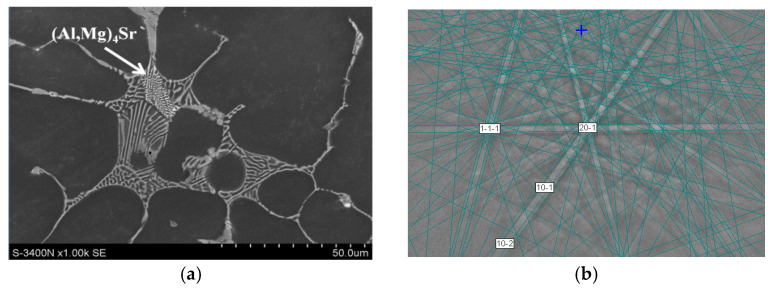
(Al,Mg)_4_Sr phase in AXJM51 alloy (**a**); indexing of pattern as Al_4_Sr phase, MAD = 0.74°, orientation = (158.5; 93.9; 29.4), crystallographic plane $(\overset{-}{5}\overset{-}{3}0)$ (**b**).

**Figure 7:**
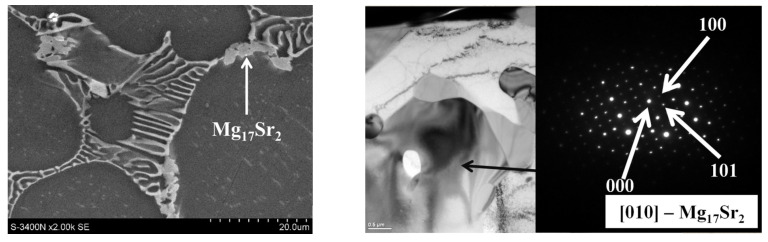
The massive Mg_17_Sr_2_ phase in AXJM53 alloy and SAED pattern of this compound (TEM), B II [-12-10].

Minor amounts of Sr dissolve in Ca-rich phases (Table 2) and form two types of intermetallic compounds. Detailed phase identification and microanalysis of the chemical composition showed the presence of the (Al,Mg)_4_Sr phase forming a lamellar eutectic with a solid solution of α-Mg (Figure 6), in which Ca can dissolve up to about 3 at.% (Table 2). In addition to the (Al,Mg)_4_Sr phase, in Mg-Al-Ca-Sr alloys there is another phase with a high Sr content, which is characterized by an irregular morphology (hereinafter, this phase will be described as the “massive phase”) and is identified as the Mg_17_Sr_2_ compound (Figure 7).

**Figure 8:**
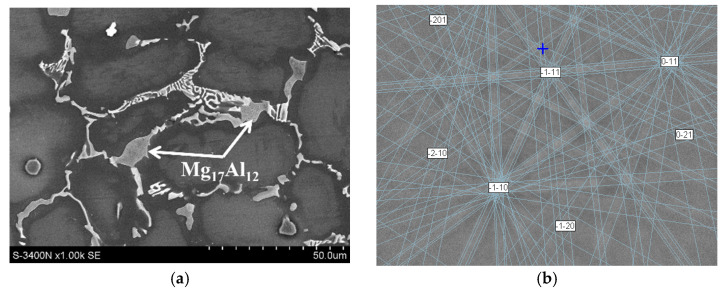
Microstructure of AXJM91 alloy with Mg_17_Al_12_ phase and supersaturated areas of α-Mg solid solution (darker areas) (**a**); indexing of pattern as Mg_17_Al_12_ phase, MAD = 0.41°, orientation = (351.2; 193; 25)°, crystallographic plane $\left(\overset{-}{6\;}\overset{-}{2\;}\overset{-}{1}\right)$ (**b**).

The Mg_17_Al_12_ phase, which forms a partially divorced eutectic with the α-Mg solid solution, was also identified (Figure 8). Low amounts of Ca (up to 1 at.%) also dissolve in this intermetallic compound. In terms of creep resistance, the presence of Ca in the crystal structure of the Mg_17_Al_12_ phase may be beneficial due to the increase in its stability at elevated temperatures [25].

**Figure 9:**
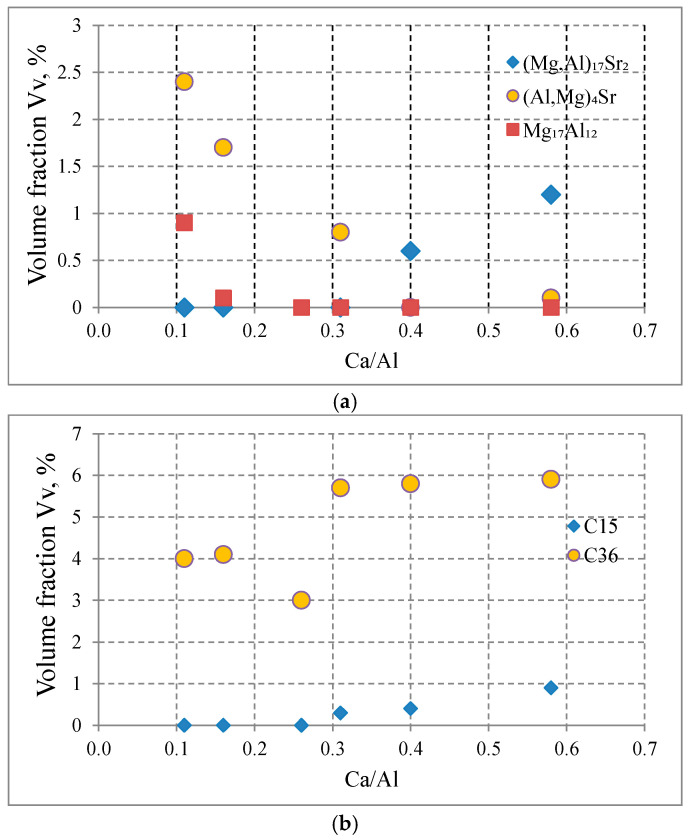
The influence of the Ca/Al ratio on the content of Sr-rich and Mg_17_Al_12_ phases (**a**) and content of C15 and C36 phases (**b**) in Mg-Al-Ca-Sr alloys.

Figure 9 and Table 3 show the influence of the Ca/Al ratio on the volume fraction of intermetallic phases and the content of Al dissolved in the α-Mg solid solution. For a Ca/Al value lower than 0.16, the Mg_17_Al_12_ phase, the Al-rich α-Mg solid solution, the C36-type (Al,Mg)_2_Ca phase, and the (Al,Mg)_4_Sr phase are observed in the microstructure. As the Ca/Al ratio increases, the volume fraction of the Mg_17_Al_12_ phase and the Al content in α-Mg decrease, and the volume fraction of the C36-type phase and the massive phase (Mg,Al)_17_Sr_2_ increase. Obviously, this is related to the presence of Ca and Sr, which have a greater chemical affinity to Al than to Mg. Therefore, phases from the Al-Ca and Al-Sr systems are formed first during the solidification of the alloy, then the concentration of aluminum in liquid metal is reduced and the Mg_17_Al_12_ phase cannot be formed at a lower temperature.

In all the tested alloys, the dominant intermetallic phase is the C36-(Al,Mg)_2_Ca phase (Table 3). It is also worth noting that the increase in the Ca/Al ratio affects the content of Sr-rich phases, causing a decrease in the content of the (Al,Mg)_4_Sr phase and an increase in the content of the (Mg,Al)_17_(Sr,Ca)_2_ phase due to the lower formation temperature of the massive phase.

**Figure 10:**
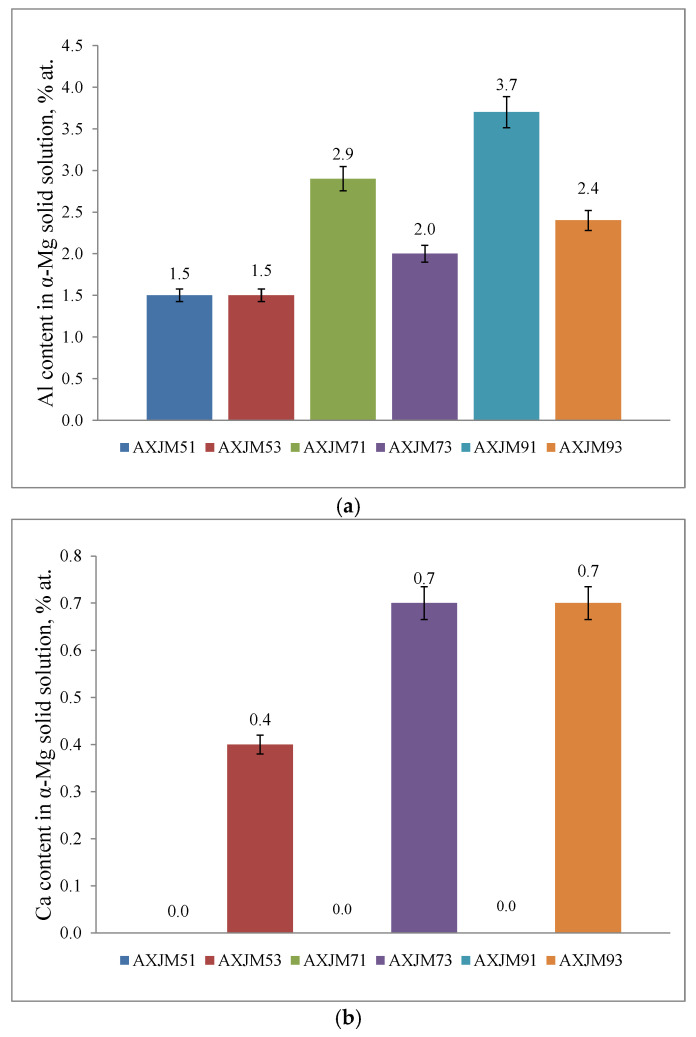
Al content (**a**) and Ca content (**b**) in the α-Mg solid solution of the tested alloys. Results regarding chemical composition in the center of α-Mg grains.

**Figure 11:**
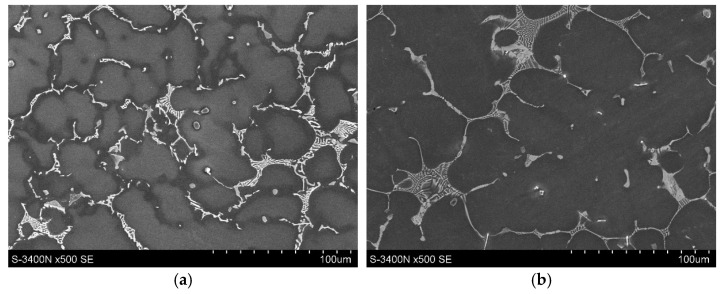
Microstructure of AXJM91 alloy with areas (darker areas) of α-Mg solid solution consisting of up to 9 at.%. Inside dendrites, the average Al content is 3.7 at.% (**a**). AXJM51 alloy, in which the strong microsegregation of Al in α-Mg was not observed (**b**).

In addition to the phase composition, an important role in the strengthening of the tested alloys is also played by the presence of alloying elements in the α-Mg solid solution. Reducing the Ca/Al ratio favors an increase in the content of Al dissolved in α-Mg (Figure 10) due to the lower content of Al, which is consumed by the Ca-rich intermetallic phases formed during crystallization. Although the gravity casting process is characterized by relatively slow cooling rates, the content of Al dissolved in α-Mg exceeds the maximum solubility at ambient temperature (1.1 at.% [26]). In alloys with a low Ca/Al ratio (Mg-9Al-1Ca-0.8Sr), strong Al microsegregation was found in the dendrites of the α-Mg solid solution (Figure 11). The high content of Al (even 9 at.%) in α-Mg favors the precipitation processes of the Mg_17_Al_12_ phase at elevated temperatures.

**Figure 12:**
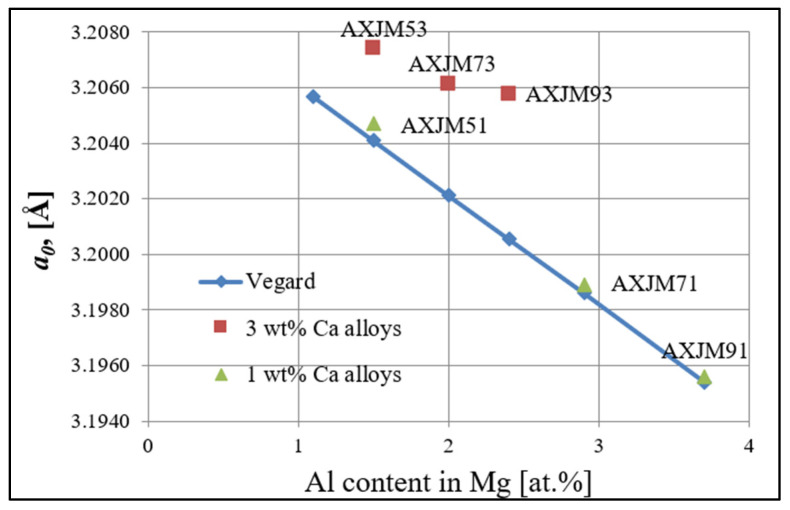
Results of the measurements of the crystal lattice parameters of the α-Mg matrix as a function of the aluminum content dissolved in the α-Mg solid solution (the graph shows only the results for the a_0_ parameter; the nature of changes for the c_0_ parameter is similar).

Both Ca and Sr exhibit very low maximum solubility in magnesium, 0.3 at.% and 0.5 at.%, respectively [27,28,29,30], which additionally decreases with lowering temperature. The results of the microanalysis of the chemical composition using an electron beam with a diameter of several nanometers (EELS and TEM) show that in the AXJM53, AXJM73, and AXJM93 alloys, Ca not only participates in the formation of intermetallic phases but also dissolves in the α-Mg solid solution. Confirmation of this can also be found in the changing lattice parameters of the α-Mg solid solution (Figure 12). In alloys containing 1 wt% Ca, the lattice parameter *a_0_* correlates quite well with Vegard’s rule (blue line) for binary Mg-Al alloys. In the case of alloys with 3 wt% Ca, the lattice parameter *a*_0_ is larger, which is related to the expansion of the crystal lattice caused by the presence of Ca (as well as Sr) atoms with a larger atomic radius (Ca—197.4 pm; Sr—215.1 pm) than Mg atoms (159.9 pm).

**Figure 13:**
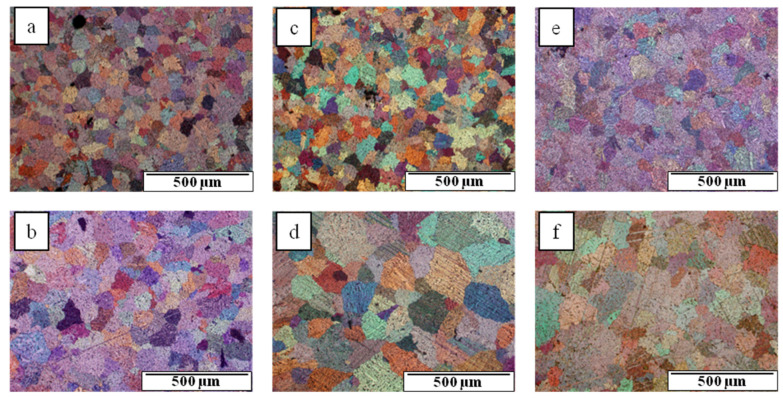
Microstructures with revealed grains of α-Mg solid solution in AXJM51 (**a**), AXJM53 (**b**), AXJM71 (**c**), AXJM73 (**d**), AXJM91 (**e**), and AXJM93 (**f**) alloys.

It seems that increasing the Ca in Mg-Al-Ca-Sr alloy content contributes to the obtainment of a coarse-grained structure (Figure 13, Table 3), although the results are not conclusive, and for some batches of castings, such a trend was not observed. There is also no clear opinion in publications regarding the influence of Ca on α-Mg grain size [31,32,33,34,35,36,37,38]. The discrepancies concern both the effect on grain size and the critical Ca content causing the formation of a fine-grained structure. In [33,34,35,39], it was found that Ca strongly refines the grain, and in [36,37,40], significant effects were not reported. Grain refinement caused by the addition of Ca is the result of heterogeneous nucleation on particles of the Al_2_Ca (C15) phase [31], as well as the result of microsegregation of alloying elements inside the α-Mg solid solution grains [38]. The critical content of Ca needed to obtain the maximum effect of fine α-Mg grains may vary within the range of 0.4–1 wt%. It is found that the most favorable results occur at 0.4 wt% [38,39,40,41] or 1 wt% [42], and a further increase in the Ca content does not affect the grain size of the α-Mg solid solution. On the other hand, exceeding the critical concentration of Ca results in the formation of a coarse-grained structure; however, the literature data differ among studies. It has been noted that significant grain growth will occur when the Ca content in the Mg-Al alloys exceeds 0.8% wt% [31], 2.0 wt% [43], or 4.0 wt% [44]. The reasons for the differing results are primarily the metallurgical purity of the alloys, as well as methodological errors consisting in the incorrect interpretation of the grain diameter and the diameter of dendritic cells. Therefore, the method of preparing metallographic specimens in order to reveal grain boundaries is important, which requires proper etching and observation using polarized light in light microscopy or the EBSD method. It seems that, in the tested alloys, the content of Al dissolved in the α-Mg solid solution will have a large impact. In [41], it was shown that the increase in the content of Al dissolved in a solid solution strongly affects the refinement of the microstructure, which results from the high tendency of this element to undergo microsegregation. Another factor conducive to the formation of a coarse-grained microstructure may be the formation of the (Al,Mg)_2_Ca phase of the C36 type instead of the Al_2_Ca phase of the C15 type, which in alloys with a low Ca content (below 0.5% Ca) is a heterogeneous crystallization nucleus.

### 3.2. Mechanical Properties of Mg-Al-Ca-Sr Alloys

The mechanical properties of the Mg-Al-Ca-Sr alloys, as well as those of the reference EZ33 alloy casted into sand molds, are presented in Table 4. AXJM71 and AXJM91 alloys show the best strength properties at ambient temperature. Alloys containing 3 wt% Ca have a higher hardness compared to the EZ33 alloy, as well as higher tensile strength and comparable ductility.

In Mg-Al-Ca-Sr alloys with an Al content above 7 wt%, an increase in the Ca content from 1 to 3 wt% reduces the tensile strength due to grain growth, an increase in the volume fraction of brittle intermetallic phases, and a decrease in the aluminum content in the α-Mg solid solution. In alloys containing 5 wt% Al, increasing the calcium content does not affect the tensile strength. The increase in the volume fraction of the brittle, eutectic phase and grain growth is compensated by the presence of plate-like Al_2_Ca precipitates inside the grains of the α-Mg solid solution.

Based on the microhardness measurements of the α-Mg solid solution (Table 4, HV0.025), the effectiveness of solution and precipitation hardening with the Al_2_Ca (C15) lamellar phase was determined. In Mg alloys containing 5 wt% Al (AXJM51 and AXJM53), the Al content in α-Mg is about 1.5 at.%. Increasing the calcium content from 1 wt% to 3 wt% increases the microhardness of the α-Mg solid solution by about 11%. This is due to the appearance of plate-like precipitates of the C15 phase. In Mg alloys containing 1 wt% Ca, plate-like precipitates in the α-Mg solid solution were not found, while the Al content in α-Mg increased from 1.5 at.%. (Mg-5Al-1Ca-0.8Sr) up to 3.7% at. (Mg-9Al-1Ca-0.8Sr). This increases the hardness of the solid solution by more than 20%. It can therefore be concluded that, in the Mg-Al-Ca-Sr alloys, Al dissolved in α-Mg strengthens the alloy more effectively than the lamellar precipitates of the Al_2_Ca (C15) phase. The slight influence of the plate-like precipitates of the Al_2_Ca phase on the strengthening of the Mg-Al-Ca-Sr alloys is related to its low volume fraction and the crystallographic relationship of the {111}_C15_‖(0001)_α-Mg_ [01$\overset{-}{1}$]_C15_‖[0$\overset{-}{1}$10]_α-Mg_. Such a relationship causes the plate-like precipitates lying in the basic plane of the hexagonal lattice of the α-Mg solid solution not to be effective in blocking the movement of dislocations in the primary slip system of the A3 crystal lattice [45].

### 3.3. Creep Properties of Mg-Al-Ca-Sr Alloys

**Figure 14:**
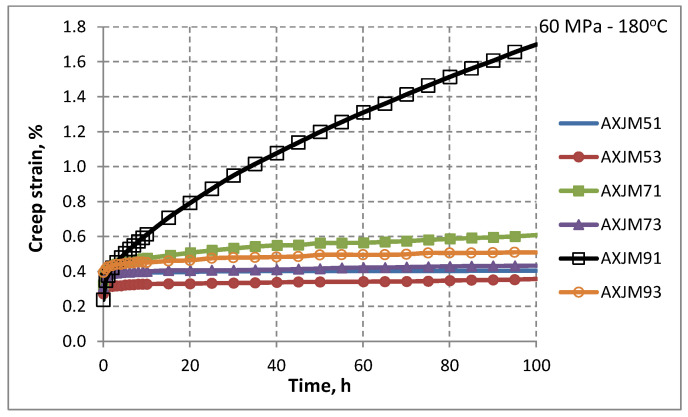
Creep curves of the Mg-Al-Ca-Sr alloys obtained at a temperature of 180 °C and at a stress of 60 MPa.

The potential application of Mg-Al-Ca-Sr alloys includes loaded elements of aircraft engines that can operate up to a temperature of about 180 °C. For this reason, creep resistance testing is of great importance. The creep curves of the AXJM alloys obtained at stresses of 60 MPa and 75 MPa at 180 °C are shown in Figure 14 (all results obtained in the creep tests are presented in Table 5). Two stages of creep can be distinguished for all curves: primary creep, in which the creep rate decreases, and steady-state creep, in which the strengthening and recovery processes are balanced. At a stress of 90 MPa, the third stage of creep is also observed, characterized by the formation of microcracks at the grain boundaries and at the interface phase and their rapid growth, which leads to the cracking of samples.

To assess the creep resistance of magnesium alloys determined in short-term creep tests (100–120 h), it is assumed that satisfactory creep resistance is obtained when the creep rate is in the order of 10^−9^ 1/s and the creep strain does not exceed 1% [5]. It should be emphasized that the steady-state creep rate is the most important feature characterizing the material’s resistance to creep and enables the design of elements with the desired durability. Obviously, in industrial conditions, these values may vary depending on the use of these alloys.

Ca has a beneficial effect on creep resistance of sand-cast Mg-Al alloys, similar to that in die-cast alloys [5]. Alloys consisting of 3 wt% Ca show very good creep resistance (low creep rates and creep strain) up to 200 °C and at a stress not exceeding 75 MPa. The improvement in creep resistance is also visible in alloys containing 1 wt% Ca; however, it is not sufficient for industrial applications.

For most metallic materials, at a creep temperature above approximately 0.3T_M_ (T_M_—melting point), thermally activated processes (climbing and sliding dislocations and diffusion) are initiated. In the case of the tested alloys, the temperature of the creep tests was close to 0.3T_M_ (the solidus temperature of the tested alloys is in the range of 429–513 °C, and the liquidus temperature is in the range of 596–613 °C). Therefore, it was expected that Mg-Al-Ca-Sr alloys would strain-harden due to the increase in dislocation density and that the creep rate would decrease. However, the results of the creep tests (Figure 14) indicate the occurrence of steady-state creep, in which the dislocation density growth processes are balanced by the recovery processes. Therefore, the power law of creep and the Arrhenius Equation (1) were used to describe the research results in order to determine the dominant creep mechanisms [5]:

(1) $$\dot{\varepsilon }=A\sigma^{n}\exp\left(\frac{-Q_{c}}{RT}\right)$$

where *A* is the constant, *R* is the gas constant, *σ* is the creep stress, *n* is the stress exponent, *Q_c_* is the activation energy, and *T* is the temperature.

**Figure 15:**
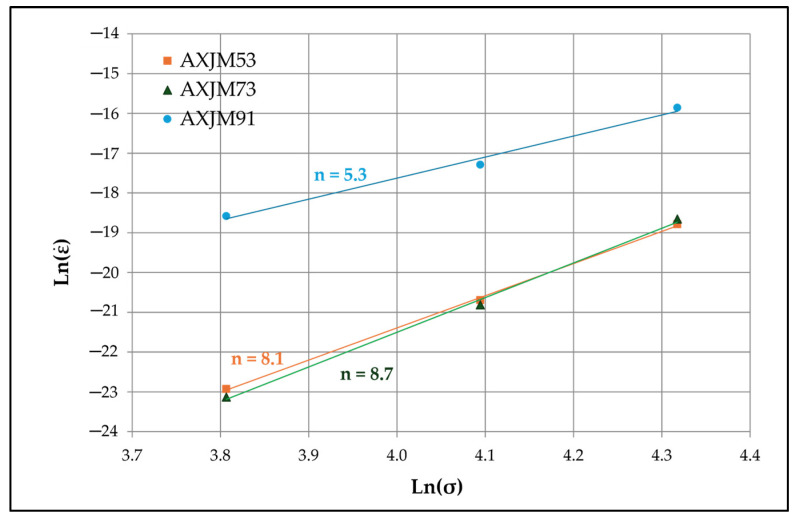
Relationship between creep rate and stress for selected Mg-Al-Ca-Sr alloys at 180 °C and in the stress range of 45–90 MPa.

Based on the knowledge of the *n* and *Q_c_* parameters, the dominant mechanisms of deformation during the creep process can be identified. For the AXJM91 alloy (Figure 15) in the stress range of 45–75 MPa at 180 °C, the power exponent *n* is 5.3, indicating the dislocation climbing as the main strain mechanism. Due to the large grain size of the α-Mg solid solution in the AXJM91 alloy (d ≈ 47 µm), the contribution of grain boundary slip to the total deformation should be insignificant. For alloys with a higher Ca content (AXJM53 and AXJM73), the power exponent *n* values are 8.1 and 8.7, which may be related to the presence of fine precipitates of the Al_2_Ca phase inside the grains of the α-Mg solution. These *n* values also indicate the dominant role of dislocation climbing in the creep deformation in the range of 45–75 MPa and at a temperature of 180 °C. It has often been demonstrated that, for magnesium alloys containing dispersed precipitates within grains, the values of the exponent *n* are in the range of 7–9 [5,46]. The presence of stable, dispersed phase particles of high hardness, constituting obstacles limiting the movement of the dislocations, and the dislocation can overcome these obstacles by climbing over long distances.

In alloys in which a higher value of the *n* exponent was observed, a greater increase in dislocation density Δρ after 100 h of creep at 180 °C was also found (Table 6). It may be due to the presence of plate-like Al_2_Ca precipitates, which, by blocking the movement of dislocations, generate new dislocations according to the Frank–Reed mechanism.

The activation energy *Q_c_* is a useful indicator that allows us to determine the creep rate controlling mechanisms. Depending on temperature and stress in metallic materials, dislocation creep (0.3Tt < T < 0.6Tt) and diffusion creep (T > 0.6Tt) can occur [47]. The results of many studies on the creep of magnesium and its alloys [47,48,49,50,51,52,53,54] have allowed the determination of values of creep activation energy for diffusion and dislocation processes. For diffusion processes, the values of *Q_c_* are as follows: Al diffusion in Mg (*Q_c_* ≈ 142–147 kJ/mol), lattice diffusion (*Q_c_* ≈ 135 kJ/mol), grain boundary diffusion (*Q_c_* ≈ 60–80 kJ/mol), pipe diffusion (*Q_c_* ≈ 92 kJ/mol), and discontinuous precipitation of the Mg_17_Al_12_ phase (*Q_c_* ≈ 22–30 kJ/mol). For dislocation processes, the values of *Q_c_* are as follows: dislocation climbing (*Q_c_* ≈ 135 kJ/mol) and transverse slip (*Q_c_* ≈ 200 kJ/mol). For grain boundary slip, *Q_c_* values are in the range of 40–60 kJ/mol.

**Figure 16:**
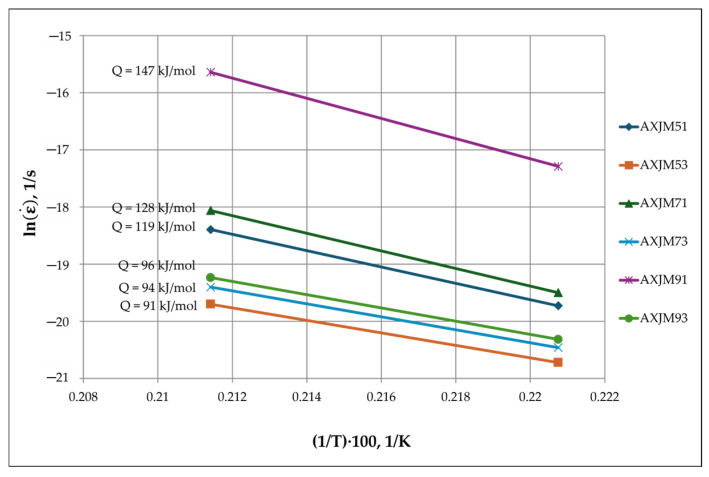
The relationship between the creep rate and the temperature of the tested alloys of Mg-Al-Ca-Sr at a stress of 60 MPa in the temperature range of 180–200 °C.

In alloys containing 3% Ca, the activation energy for steady-state creep is in the range of 91–96 kJ/mol (Figure 16), which suggests that plastic deformation during creep is controlled by pipe diffusion. Higher values of the activation energy *Q_c_* were obtained for alloys containing AXJM51 and AXJM71 (119–128 kJ/mol), indicating that the creep deformation is mainly controlled by the dislocation climbing. In the case of the AXJM91 alloy (*Q_c_* = 147 kJ/mol), the Al diffusion in the α-Mg solid solution or the transverse slip of the edge dislocations makes a greater contribution to the creep deformation.

In magnesium alloys, especially die-cast alloys, grain boundary slip also plays an important role in the creep conditions. In studies on die-cast Mg-Al-Ca-Sr alloys [55], i.e., with fine-grained structure (grain diameter d ≈ 10 µm), it was shown that, at a temperature of 175 °C in the stress range of 40–70 MPa, creep is controlled by grain boundary slipping. In the case of Mg-Al-Ca-Sr alloys after sand casting, the obtained results indicate that grain boundary slip plays a rather marginal role. This may be related to a much larger grain size (d ≈ 40–60 µm) of sand-cast alloys and thus a smaller surface of grain boundaries. Moreover, a continuous skeleton of hard and stable intermetallic compounds will contribute to reduction in the grain boundary creep.

### 3.4. Influence of Microstructure on Creep Properties of Mg-Al-Ca-Sr Alloys

**Figure 17:**
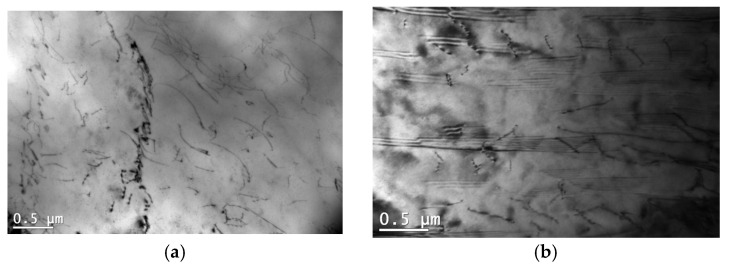
Dislocations (**a**) and stacking faults (**b**) in sand-cast AXJM53 alloy.

**Figure 18:**
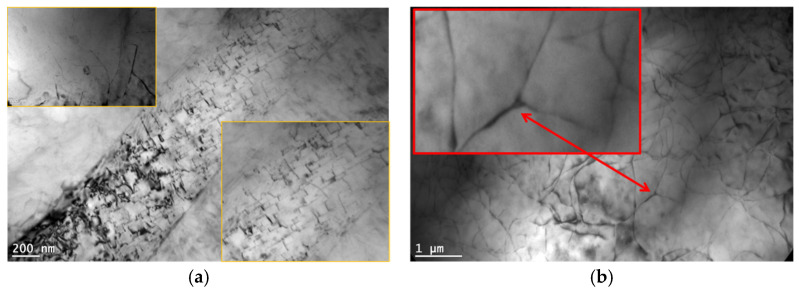
Dislocation substructure in AXJM91 alloy (**a**) and split dislocations in AXJM53 alloy (**b**) suggest the occurrence of cross-slip after creep at 180 °C and at 60 MPa for 100 h.

Observations conducted using a transmission electron microscope were used to understand the deformation behavior of the alloys at elevated temperatures. The tested alloys in the as-cast state are characterized by high dislocation density (ρ ≈ 10^−13^ [1/m^2^] (Table 6) and the presence of stacking faults (Figure 17). The tendency to form a cellular dislocation structure is slight in magnesium alloys due to low stacking fault energy (SFE); therefore, after creep, the split dislocations are mainly visible in the microstructure, indicating the cross-slip mechanism that occurs when a dislocation changes its slip plane to a different crystallographic plane (Figure 18). This mechanism of dislocation movement is observed in metals when the creep temperature is lower than 0.5Tt and the value of the creep activation energy *Q_c_* is lower than the value of the self-diffusion activation energy (for Mg, *Q_SD_* = 135 kJ/mol) [47].

**Figure 19:**
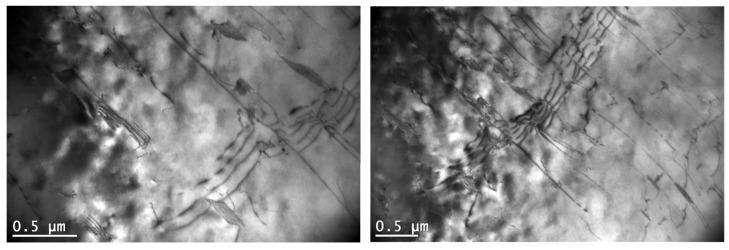
AXJM71 alloy with visible <a> and <a + c> dislocations that indicate climb and cross slip of dislocations in AXJM71 alloy after creep at 180 °C and at 60 MPa for 100 h.

Dislocations moving both in the basic slip system $\left\langle 11\overset{-}{2}0\right\rangle$ (0001) and in the pyramidal slip system $\left\langle 11\overset{-}{2}0\right\rangle$ {10-$\overset{-}{1}$1} can be observed in samples crept at 180 °C (Figure 19), indicating the significant contribution of edge dislocation climbing to the creep deformation of alloys containing 1% Ca. In alloys with a higher calcium content (3% Ca), dislocation climbing is also observed, but the elongation of these alloys in creep tests is clearly lower compared to Ca-poor alloys (Table 5). According to the activation energy calculations, the pipe diffusion dominates in the overall deformation, contributing to dislocation climbing.

**Figure 20:**
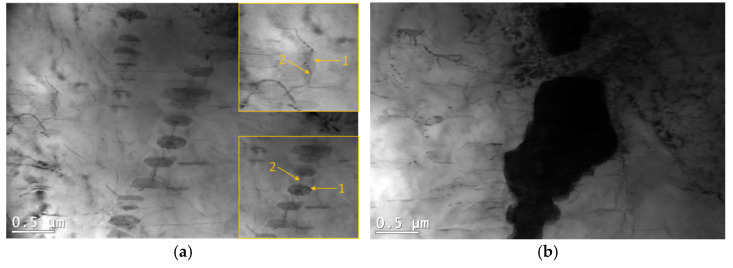
Blocking of the movement of dislocation motion plate-shaped precipitates of the Al_2_Ca (C15) phase during creep at 180 °C at 60 MPa in AXJM53 (**a**) and accumulation of dislocations in the vicinity of (Al,Mg)_2_Ca (C36) in the AXJM53 alloy after creep at 180 °C and at 60 MPa for 100 h (**b**). 1—precipitate, 2—bent dislocation.

A significant influence on the creep resistance of Mg-Al-Ca-Sr alloys containing 3% Ca is exerted by plate-like Al_2_Ca-C15 precipitates located in the basic crystallographic planes (0001) of the α-Mg matrix. Their effect on blocking the movement of dislocations in the slip plane is not significant at ambient temperature, but at elevated temperatures, where additional slip systems are activated and the movement of dislocations is possible in prismatic and pyramidal planes, effects (avoiding precipitates and cross slip) indicating blocking of dislocation movement by C15 precipitates are clearly observed (Figure 20).

In the vicinity of intermetallic phases forming eutectics (C36, C14, (Al,Mg)_4_Sr, and (Mg,Al)_17_Sr_2_) characterized by high melting point and high hardness, dislocation accumulations were found (Figure 20b). At ambient temperature, a large accumulation of dislocations favors the nucleation of cracks at the interface, which results in low tensile strength. At temperatures above 150 °C, dynamic recrystallization may occur in these areas, and crack formation may be delayed.

**Figure 21:**
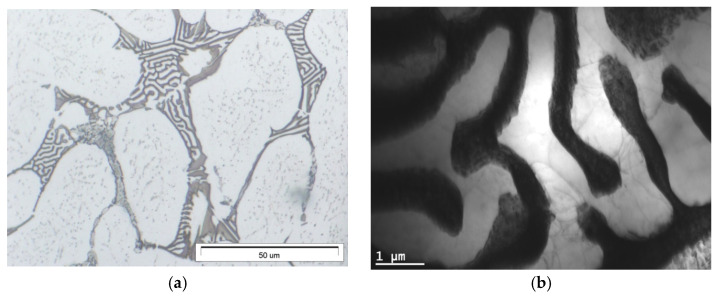
Microstructure of AXJM73 alloy after creep at 180 °C and at 75 MPa for 100 h (**a**); microstructure of AXJM53 alloy after creep at 180 °C and at 60 MPa for 100 h (**b**).

Observations of the microstructure of the tested alloys after creep tests at a temperature of 180 °C and a stress range of 60–75 MPa showed no significant changes in the morphology of the phases (Al,Mg)_2_Ca-type C36, (Mg,Al)_17_Sr_2_, and (Al,Mg)_4_Sr forming eutectics with α-Mg solid solution (Figure 21). Analysis of the phase composition using methods with the use of electron diffraction (TEM) and X-ray diffraction (XRD) exhibited no changes in the phase composition of the crept alloys. This indicates the high stability of the intermetallic phases (Al,Mg)_2_Ca-type C36, (Mg,Al)_17_Sr_2_, and (Al,Mg)_4_Sr at a temperature of 180 °C. The high thermal stability of the intermetallic phases forming eutectics was also confirmed after long-term annealing (1000 h) at 200 °C.

**Figure 22:**
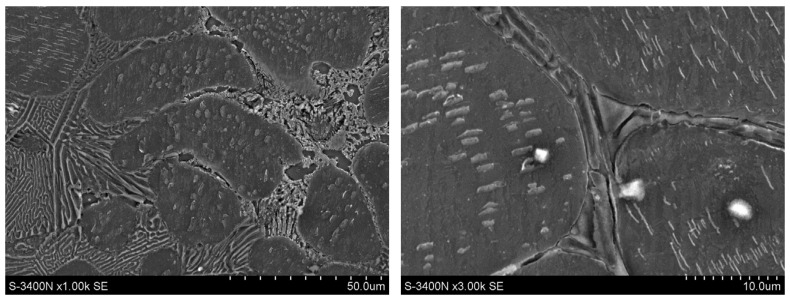
Microstructure of AXJM53 alloy after creep at 180 °C and at 75 MPa for 100 h after strong etching in 3% HNO_3_ solution in alcohol.

While eutectic phases are stable, microstructural changes occur inside the grains of the α-Mg solid solution. After creep tests were performed on the AXJM53 alloy at a temperature of 180 °C for 100 h, a higher volume fraction of lamellar Al_2_Ca phase precipitations (V_v_ = 1.4%) was found inside the grains of the α-Mg solid solution compared to the as-cast alloy (V_v_ = 0.9%) (Table 7) due to the precipitation process of the plate-like Al_2_Ca (C15) phase from the α-Mg solid solution. Moreover, the initial stages of spheroidization of the C15 precipitates are visible (Figure 22), which will contribute to faster destruction of alloys under creep conditions. Precipitation of this phase was also observed after creep at 180 °C in other Mg-Al-Ca-Sr alloys containing 3 wt% Ca.

**Figure 23:**
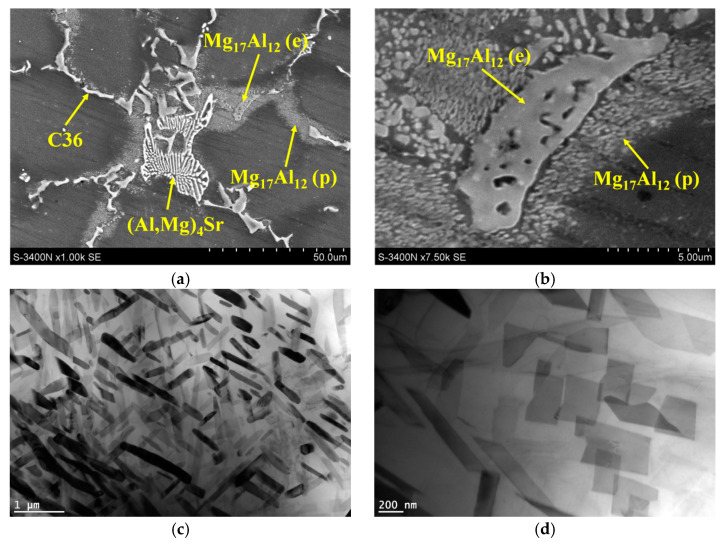
Microstructure of AXJM91 alloy after creep at 180 °C and at 75 MPa for 100 h with eutectic Mg_17_Al_12_ (e) phase and plate-shaped precipitates of Mg_17_Al_12_ (p) phase arising in supersaturated areas of α-Mg solid solution surrounding eutectics, SEM (**a**,**b**); plate-shaped precipitates of Mg_17_Al_12_ phase (**c**,**d**).

In the AXJM91 and AXJM71 alloys crept at a temperature of 180 °C, plate-like precipitates of the Mg_17_Al_12_ phase were observed in the supersaturated areas of the α-Mg solid solution surrounding the eutectics (Figure 23). This leads to the depletion of the α-Mg solid solution in aluminum. Obviously, the content of the precipitated Mg_17_Al_12_ phase is lower in the AXJM71 alloy in comparison to the AXJM91 alloy due to the lower Al amount dissolved in the α-Mg matrix.

**Figure 24:**
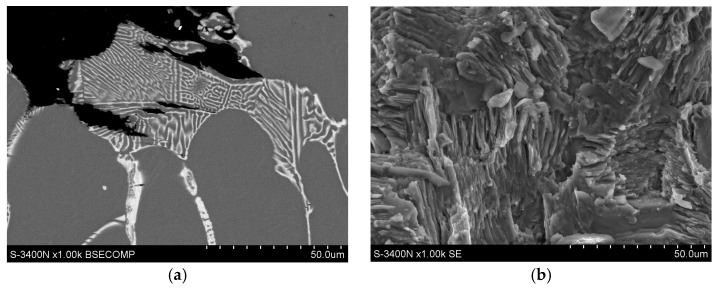
Cracks occurring during creep of the AXJM53 alloy at 180 °C at 90 MPa (**a**). The propagation of the cracks occurs mainly along the precipitations of the (Al,Mg)_2_Ca (C36) phase (**b**).

**Figure 25:**
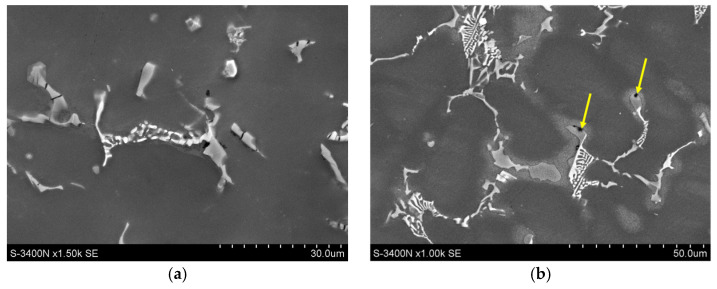
Cracks occurring during creep of the AXJM71 alloy at 180 °C at 75 MPa (**a**); voids appearing at the interface α-Mg/Mg_17_Al_12_ in the AXJM91 alloy after creep at 180 °C at a stress of 75 MPa for 100 h (**b**).

The destruction of the tested alloys during creep is caused primarily by the initiation of cracks at the interfaces and the propagation of these cracks (Figure 24). As the creep strain increases, the stress concentration on the precipitates increases, which leads to their cracking and loss of connection with the alloy’s matrix. In Mg-Al-Ca-Sr alloys containing 3 wt% Ca, the first cracks are observed mainly at the α-Mg/C36 interface. In the AXJM91 alloy, creep cracks are initiated at the interface between the α-Mg solid solution and the Mg_17_Al_12_ phase (Figure 25b).

It is well known that the Mg_17_Al_12_ phase adversely affects creep resistance, which is confirmed by the obtained test results. The precipitation processes of this phase during creep cause a decrease in the Al concentration in the solid solution, facilitating lattice diffusion and further contributing to the reduction in creep resistance. Therefore, the creep resistance of the AXJM91 alloy is much lower than that of the other tested alloys.

The formation of cracks at the α-Mg/C6 interface is related to the concentration of dislocations, the high hardness of the C36 phase, and the low ability to accommodate plastic deformation. The lamellar structure of this phase will contribute to slower crack development, and it seems that the presence of this phase is more advantageous than the presence of massive phases such as (Mg,Al)_17_(Sr,Ca)_2_.

### 3.5. Comparison of Mg-Al-Ca-Sr Alloys with EZ33 Magnesium Alloy

**Figure 26:**
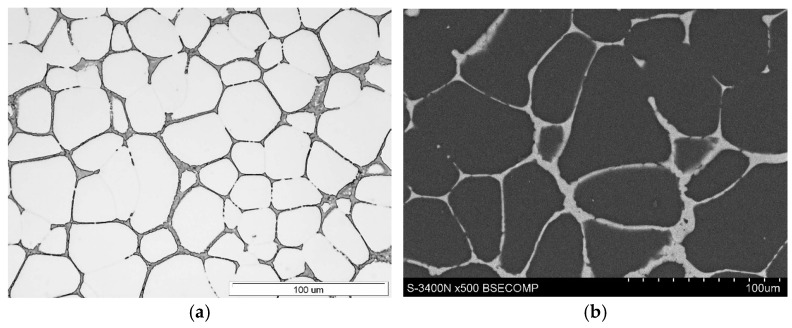
Microstructure of as-cast EZ33 magnesium alloy: LM (**a**) and SEM (**b**).

The microstructure and mechanical properties of the as-cast EZ33 magnesium alloy have been extensively described [9,18,19,20]. The typical microstructure of this alloy after gravity casting into sand molds consists of α-Mg solid solution dendrites and a (Mg,Zn)_12_RE (Mg_12_Nd type) compound at interdendritic areas (Figure 26). This intermetallic compound exhibits good stability of morphology and chemical composition up to 250 °C. Solution strengthening caused by the presence of Zn atoms in the α-Mg solid solution and the presence of the thermally stable (Mg,Zn)_12_RE phase strengthens the grain boundaries and contributes to the obtainment of quite satisfactory mechanical properties at ambient temperature (Table 8) and good creep resistance at temperatures up to 200 °C and stresses up to 60 MPa (Table 9). It is worth noting that the mechanical properties of sand castings are about 10% lower compared to alloy casts into permanent molds [9]. Therefore, the EZ33 alloy can be used for elements operating at elevated temperatures (up to 200 °C) in the aviation and automotive industries. Mechanical properties can be slightly improved by applying T5 heat treatment consisting of annealing at 170–200 °C for 10–16 h with cooled air due to the formation of Zn_2_Zr_3_ precipitates inside the α-Mg solid solution.

The most advantageous mechanical properties among the tested alloys are those of the AXJM93 alloy, while the AXJM53 and AXJM73 alloys also demonstrate the required creep resistance up to 200 °C and acceptable strength properties at ambient temperature. Mg-Al-Ca-Sr alloys with a 3 wt% Ca content have another advantage: they contain lighter alloying elements than the EZ33 alloy; hence, the specific strength is more advantageous (Table 10). In the case of AXJM53 and AXJM73 alloys, the disadvantage is the relatively low elongation. Increasing the Al content to 9% contributes to the obtainment of plastic properties similar to those of the EZ33 alloy. It should be emphasized that the use of Mg-Al-Ca-Sr alloys in aviation requires additional tests, including those for fatigue strength, and possibly requires applying protective coatings against corrosion.

The advantage of using Mg-Al-Ca-Sr alloys, apart from the lower price of alloying elements, is the casting process at a lower temperature. In the case of the EZ33 alloy, melting is carried out at a temperature of 780–815 °C, while for Mg-Al-Ca-Sr alloys, it is possible to carry out the process at a temperature of 730 °C. The time of preparation of the liquid alloy in both cases is similar. Other advantages related to melting Mg-Al-Ca-Sr alloys include the non-flammability of these alloys during melting (Mg has a very low ignition temperature), which makes the casting process safer. However, it is necessary to use protective atmospheres in order to obtain the desired chemical composition of the alloy and to avoid an excessive number of non-metallic inclusions.

**Figure 27:**
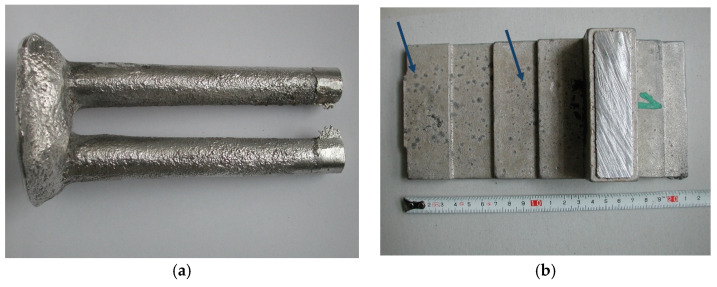
AXJM53 alloy after casting in an induction furnace using a mold made of bentonite mass (**a**); AXJM53 alloy after casting in industrial conditions using an electric furnace and a mold made of furan mass (**b**).

Despite the many advantages of Mg-Al-Ca-Sr alloys, mold sticking and hot cracking can be a serious problem during the casting process. This problem was mainly observed during die casting of Mg-Al-Ca alloys or gravity casting into metal molds and most frequently concern Mg-Al alloys with a low calcium content (up to approx. 1–2% Ca). At higher Ca contents, susceptibility to hot cracking and the formation of sticking is not greater than in typical magnesium alloys (AM50 and AZ91) [56,57,58,59]. No problems were found in the case of Mg-Al-Ca-Sr alloys gravity-cast into sand molds. After casting in an induction furnace into a mold based on quartz bentonite mass, the surface of the castings was free of sticking and cracks, and the surface quality was of a satisfactory level (Figure 27a). It is worth noting that the tests of Mg-Al-Ca-Sr alloys conducted in industrial conditions with melting in an electric furnace and casting into a mold based on furan mass showed the absence of hot cracks and a high susceptibility to the formation of sticking, regardless of the chemical composition of the Mg-Al-Ca-Sr alloys. The sticking was easily removed after sand blastin; however, staining remained visible (Figure 27b). The surface quality of the Mg-Al-Ca-Sr alloy after casting in industrial conditions was clearly worse compared to that of the EZ33 alloy, but it can be improved by an appropriate selection of molding materials.

## 4. Conclusions

The aim of the research was to develop a magnesium alloy consisting of cheap alloying elements, which after gravity casting into sand molds will be characterized by mechanical properties similar to those of the EZ33 alloy at temperatures up to 200 °C. The aim was achieved by appropriate selection of alloying elements such as Al, Ca, and Sr. This allowed us to obtain alloys that exhibit the presence of a skeleton of intermetallic phase precipitates stable at elevated temperature, strengthened α-Mg solid solution by alloying elements (especially Al), and plate-like precipitates of the C15 Laves phase inside the α-Mg solid solution.

Based on the research conducted and analysis of the results, the following conclusions were formulated:

1. Increasing the Ca concentration in Mg-Al-Sr alloys causes a decrease in the volume fraction of the Mg_17_Al_12_ phase, the degree of supersaturation of the α-Mg solid solution, and the formation of Laves Ca-rich (Mg,Al)_2_(Ca,Sr) (C36) and (Mg,Al)_2_Ca (C14) phases and Sr-rich (Al,Mg)_4_Sr and (Mg,Al)_17_Sr_2_ phases. For large Ca/Al concentration ratios, additional plate-like precipitates of the Al_2_Ca (C15) Laves phase appear inside the dendrites of the α-Mg solid solution.
2. Alloys containing 1% Ca are characterized by good mechanical properties at ambient temperature, but their creep resistance at 200 °C is insufficient. Increasing the Ca content to 3% causes a decrease in mechanical properties at ambient temperature while achieving satisfactory creep resistance at 200 °C. The most favorable mechanical properties at ambient and elevated temperatures are achieved by the AXJM93 alloy (R_m_ = 149 MPa, R_p0.2_ = 102 MPa, A = 2.5%, creep properties at a temperature of 200 °C and at a stress of 60 MPa—$\dot{\varepsilon }\;$~10^−9^ 1/s, ε = 0.64%), which can be used as a substitute for the EZ33 alloy due to its comparable mechanical properties at ambient temperature, slightly better creep resistance at temperatures up to 200 °C, and lower density.
3. Factors contributing to the improvement in the creep resistance of Mg-Al-Ca-Sr alloys at 200 °C include the following: solution strengthening caused by the dissolution of Al and Ca in α-Mg, precipitation strengthening resulting from the presence of plate-like precipitates of the Al_2_Ca (C15) phase with crystallographic orientation of the type {111}_C15_‖(0001)_α-Mg_ [01$\overset{-}{1}$]_C15_ ‖[0$\overset{-}{1}$10] _α-Mg_, and the presence of high-melting Laves phases in interdendritic areas. Conversely, the Mg_17_Al_12_ phase and the presence of supersaturated α-Mg solid solution, in which the Al content significantly exceeds the solubility limit of Al in Mg at 200 °C, exhibit an unfavorable effect on creep resistance.
4. In the case of 1% Ca alloys, dislocation climbing was found to be the dominant creep mechanism at 180 °C. In the 3% Ca alloys, the steady-state creep activation energy (*Q_c_*) values indicate that creep deformation is controlled by pipe diffusion. Grain boundary slip, which is a significant contributor to creep deformation in die-cast Mg-Al-Ca-Sr alloys, plays a marginal role in sand-cast alloys due to the larger grain size.

## Figures and Tables

**Table 1:** Chemical composition of Mg-Al-Ca-Sr and EZ33 alloys (wt%). The standard deviation is shown in brackets.

**Alloy**	**Al**	**Ca**	**Sr**	**Mn**	**Si**	**Fe**	**Mg**
AXJM51	4.98 (0.38)	1.24 (0.15)	0.75 (0.12)	0.19 (0.05)	0.22 (0.06)	0.0013 (0.002)	Balance
AXJM53	5.11 (0.19)	3.01 (0.23)	0.68 (0.22)	0.16 (0.08)	0.18 (0.07)	0.0013 (0.001)	Balance
AXJM71	6.80 (0.32)	1.22 (0.13)	0.70 (0.16)	0.13 (0.09)	0.12 (0.1)	0.0012 (0.0003)	Balance
AXJM73	7.09 (0.34)	3.05 (0.24)	0.61 (0.14)	0.10 (0.07)	0.10 (0.08)	0.0007 (0.001)	Balance
AXJM91	9.01 (0.51)	1.13 (0.24)	0.81 (0.22)	0.19 (0.08)	0.26 (0.07)	0.0011 (0.0011)	Balance
AXJM93	8.67 (0.50)	2.89 (0.28)	0.60 (0.1)	0.16 (0.07)	0.16 (0.05)	0.001 (0.001)	Balance
Alloy	Zn	RE	Zr	Mn	Fe	Ni, Cu	Mg
EZ33	2.70	3.18	0.53	0.02	0.002	<0.001	Balance

RE—mischmetal mixture 50Ce-25La-20Nd-5Pr (wt%).

**Table 2:** Results of the chemical composition microanalysis for precipitates in the tested alloys (at.%); EDS-SEM. Standard deviation is given in brackets.

Phase	Mg	Al	Ca	Sr	Mn
(Al,Mg)_2_Ca (C36)	10.0 (1.7)	57.6 (5.6)	30.4 (2.8)	2.0 (0.24)	-
(Mg,Al)_2_Ca (C14)	17.5 (2.1)	54.2 (3.7)	26.5 (2.3)	1.8 (0.19)	
(Mg,Al)_17_Sr_2_	71.8 (3.9)	19.5 (1.8)	2.5 (1.1)	6.2 (0.13)	-
(Al,Mg)_4_Sr	47.8 (3.9)	41.2 (3.9)	3.2 (1.2)	7.8 (0.25)	-
Al_2_Ca (C15)	75.2 (7.8)	16.0 (2.4)	8.8 (1.8)	-	-
Mg_17_Al_12_	70.7 (3.1)	28.5 (1.9)	0.7 (0.2)	0.1 (0.11)	-

**Table 3:** Volume fraction of intermetallic compounds and grain size of α-Mg solid solution determined by quantitative metallography and Al content in α-Mg solid solution. The standard deviation is given in brackets and, in the case of grain size, the coefficient of variation in %.

Alloy	Ca/Al Ratio	Al Content in α-Mg, at.%	α-Mg Grain Size, µm	Phase Composition	Volume Fraction, %
AXJM51	0.26	1.5 (0.18)	58.1 (19.6)	(Al,Mg)_2_Ca—C36	3.0 (0.24)
				(Mg,Al)_17_Sr_2_	2.0 (0.18)
				(Al,Mg)_4_Sr	0.1 (0.07)
				Al_8_Mn_5_	0.1 (0.08)
AXJM53	0.58	1.5 (0.16)	75.2 (34.7)	(Al,Mg)_2_Ca—C36	5.9 (0.47)
				Al_2_Ca—C15	0.9 (0.4)
				(Mg,Al)_17_Sr_2_	1.2 (0.11)
				(Al,Mg)_4_Sr	0.1 (0.03)
				(Mg,Al)_2_Ca—C14	0.9 (0.05)
				Al_8_Mn_5_	0.1 (0.02)
AXJM71	0.16	2.9 (0.25)	59.0 (25.0)	(Al,Mg)_2_Ca—C36	4.1 (0.33)
				(Al,Mg)_4_Sr	1.7 (0.1)
				Al_8_Mn_5_	0.1 (0.06)
				Mg_17_Al_12_	0.1 (0.04)
AXJM73	0.40	2.0 (0.32)	113.7 (68.9)	(Al,Mg)_2_Ca—C36	5.8 (0.41)
				Al_2_Ca—C15	0.4 (0.3)
				(Mg,Al)_17_Sr_2_	0.6 (0.04)
				(Mg,Al)_2_Ca—C14	0.3 (0.04)
				Al_8_Mn_5_	0.1 (0.01)
AXJM91	0.11	3.7 (0.55)	66.2 (25.9)	(Al,Mg)_2_Ca—C36	4.0 (0.28)
				(Al,Mg)_4_Sr	1.9 (0.12)
				Al_8_Mn_5_	0.1 (0.02)
				Mg_17_Al_12_	0.9 (0.16)
AXJM93	0.31	2.4 (0.17)	77.6 (28.9)	(Al,Mg)_2_Ca—C36	5.7 (0.45)
				Al_2_Ca—C15	0.3 (0.1)
				(Al,Mg)_4_Sr	0.8 (0.1)
				Al_8_Mn_5_	0.1 (0.06)

**Table 4:** Test results of mechanical properties of Mg-Al-Ca-Sr and EZ33 alloys at ambient temperature and 180 °C. Standard deviation given in parentheses.

Alloy	HV2	Hardness of α-Mg HV0.025	21 °C			180 °C		
			R_m_, MPa	R_p0.2_, MPa	A_5_, %	R_m_, MPa	R_p0.2_, MPa	A_5_, %
AXJM51	49(6)	45(1)	138(6)	101(5)	1.9(0.2)	107(4)	71(4)	9.8(1.2)
AXJM53	58(1)	50(2)	135(4)	104(3)	2.1(0.3)	123(8)	84(7)	3.2(0.6)
AXJM71	50(1)	53(2)	174(6)	113(5)	3.1(0.3)	119(7)	77(5)	5.5(0.7)
AXJM73	60(6)	52(2)	137(5)	105(4)	2.1(0.2)	119(3)	88(5)	2.9(0.4)
AXJM91	56(2)	56(1)	165(6)	109(6)	2.9(0.4)	102(2)	78(6)	12.5(1.8)
AXJM93	60(7)	54(1)	149(7)	102(4)	2.5(0.3)	115(7)	81(3)	3.9(0.9)

**Table 5:** Creep test results of Mg-Al-Ca-Sr alloys.

Alloy	Stress, MPa	Temp., °C	Time Test, h	Instant Strain ε_n,_ %	Creep Strain ε, %	$\mathrm{Creep}\;\mathrm{Rate}\;\dot{\varepsilon },$ 1/s
AXJM53	45	180	100	0.15	0.28	1.1 × 10^−10^
AXJM73	45	180	100	0.16	0.27	9.0 × 10^−11^
AXJM91	45	180	100	0.11	0.92	8.5 × 10^−9^
AXJM51	60	180	100	0.57	0.94	2.7 × 10^−9^
AXJM53	60	180	100	0.31	0.36	1.0 × 10^−9^
AXJM71	60	180	100	0.32	0.61	3.4 × 10^−9^
AXJM73	60	180	100	0.31	0.43	1.3 × 10^−9^
AXJM91	60	180	100	0.24	1.7	3.1 × 10^−8^
AXJM93	60	180	100	0.39	0.51	1.5 × 10^−9^
AXJM51	75	180	100	1.03	3.56	4.0 × 10^−8^
AXJM53	75	180	100	0.50	1.38	9.9 × 10^−9^
AXJM71	75	180	100	0.61	5.59	1.0 × 10^−7^
AXJM73	75	180	100	0.54	1.15	7.9 × 10^−9^
AXJM91	75	180	100	0.44	6.1	1.3 × 10^−7^
AXJM93	75	180	100	0.31	1.93	3.3 × 10^−8^
AXJM53	90	180	16	1.37	5.17	4.9 × 10^−7^
AXJM71	90	180	23	1.51	12.46	9.1 × 10^−7^
AXJM73	90	180	31	1.47	6.1	8.4 × 10^−8^
AXJM91	90	180	9	1.2	7.2	1.3 × 10^−5^
AXJM51	60	200	100	0.63	1.27	1.03 × 10^−8^
AXJM53	60	200	100	0.33	0.44	2.78 × 10^−9^
AXJM71	60	200	100	0.34	0.98	1.43 × 10^−8^
AXJM73	60	200	100	0.32	0.54	3.61 × 10^−9^
AXJM91	60	200	100	0.28	6.22	1.61 × 10^−7^
AXJM93	60	200	100	0.43	0.64	4.43 × 10^−9^

**Table 6:** Results of dislocation density measurements using the modified Warren–Averbach method (XRD) of the tested alloys in the as-cast condition and after creep at a temperature of 180 °C and a stress of 60 MPa. The standard deviation is given in brackets.

Alloy	Alloy State	Dislocation Density ρ [1/m^2^]	Δρ [%]
AXJM53	Sand-cast	2.9 × 10^13^ (1.2 × 10^12^)	21
	60 MPa/180 °C	3.5 × 10^13^ (1.9 × 10^12^)	
AXJM73	Sand-cast	3.4 × 10^13^ (2.1 × 10^12^)	18
	60 MPa/180 °C	4.0 × 10^13^ (1.7 × 10^12^)	
AXJM91	Sand-cast	9.7 × 10^13^ (2.6 × 10^12^)	3
	60 MPa/180 °C	1.0 × 10^14^ (3.7 × 10^12^)	

**Table 7:** Volume fraction of phases in Mg-Al-Ca-Sr alloys after creep at 180 °C and 60 MPa stress. Standard deviation given in brackets.

Alloy	State	(Al,Mg)_2_Ca—C36	Al_2_Ca—C15	Mg_17_Al_12_	(Mg,Al)_17_Sr_2_	(Al,Mg)_4_Sr
AXJM51	As-cast	3.0 (0.24)	-	-	2.0 (0.18)	0.1 (0.07)
	60 MPa/180 °C	3.2 (0.36)	-	-	2.1 (0.45)	0.1 (0.10)
AXJM53	As-cast	5.9 (0.47)	0.9 (0.5)	-	1.2 (0.11)	0.1 (0.03)
	60 MPa/180 °C	5.7 (0.89)	1.4 (0.5)	-	1.4 (0.11)	0.12 (0.1)
AXJM71	As-cast	4.1 (0.33)	-	0.1 (0.04)	-	1.7 (0.1)
	60 MPa/180 °C	4.2 (0.94)	-	0.4 (0.08)	-	1.7 (0.3)
AXJM73	As-cast	5.8 (0.41)	0.4 (0.3)	-	0.6 (0.04)	-
	60 MPa/180 °C	6.0 (0.92)	0.9 (0.3)	-	0.8 (0.15)	
AXJM91	As-cast	4.0 (0.28)	-	0.9 (0.16)	-	1.9 (0.12)
	60 MPa/180 °C	3.8 (0.38)	-	1.9 (0.17)	-	1.8 (0.25)
AXJM93	As-cast	5.7 (0.45)	0.3 (0.1)	-	-	0.8 (0.1)
	60 MPa/180 °C	5.9 (0.28)	0.7 (0.3)	-	-	0.9 (0.4)

**Table 8:** Mechanical properties of sand-cast EZ33 alloy (T5—heat treatment 10 h at 170 °C and cooled air).

Casting Method	Heat Treatment	R_m_ MPa	R_p0.2_ MPa	Elongation A_10_, %
Sand casting	T5	119–140	80–95	3

**Table 9:** Creep test results of sand-cast EZ33 magnesium alloy (T5—heat treatment 10 h at 170 °C and cooled air).

Alloy	Heat Treatment	Stress, MPa	Temperature, °C	Test Time, h	Creep Strain ε, %	$\mathrm{Creep}\;\mathrm{Rate}\;\dot{\varepsilon }$, 1/s
EZ33	-	60	180	100	0.89	5.0 × 10^−9^
EZ33	-	60	200	100	1.14	7.7 × 10^−9^
EZ33	-	60	225	87	5.9	2.4 × 10^−8^
EZ33	T5	60	180	100	0.64	3.6 × 10^−9^
EZ33	T5	60	200	100	0.90	6.5 × 10^−9^
EZ33	T5	75	200	100	1.91	2.4 × 10^−8^
EZ33	T5	30	250	100	0.82	1.4 × 10^−8^
EZ33	T5	40	250	100	3.3	6.9 × 10^−8^

**Table 10:** Specific strength, yield strength, elongation, and creep properties of Mg-Al-Ca-Sr and EZ33 alloys.

Alloy	Density ρ, g/cm^3^	Specific Strength R_m_/ρ, MPa∙cm^3^/g	Specific Yield Strength R_p0.2_/ρ, MPa∙cm^3^/g	Elongation, %	Creep Strain at 200 °C and 60 MPa After 100 h, %	$\mathrm{Creep}\;\mathrm{Rate}\;\dot{\varepsilon }$, at 200 °C and 60 MPa After 100 h, 1/s
AXJM51	1.81	76.4	55.9	1.9	1.27	1.03 × 10^−8^
AXJM71	1.82	95.5	62.0	3.1	0.98	1.43 × 10^−8^
AXJM91	1.85	89.2	58.9	2.9	6.22	1.61 × 10^−7^
AXJM53	1.80	75.0	57.8	2.1	0.44	2.78 × 10^−9^
AXJM73	1.82	75.4	57.8	2.1	0.54	3.61 × 10^−9^
AXJM93	1.84	81.2	55.6	2.5	0.64	4.43 × 10^−9^
EZ33 *	2.08	57.2	38.5	2.9	0.90	6.5 × 10^−9^
EZ33 **	2.08	67.3	43.3	3.0	-	-

* Own research, ** manufacturer data [9].

## Data Availability

The original contributions presented in this study are included in the article. Further inquiries can be directed to the corresponding author.
